# Students in action

**Published:** 2009-11-25

**Authors:** A Carbune

**Affiliations:** Society of Students in Medicine BucharestRomania

It was the moment when education, as we know it today, did not exist. In 1857, visionary doctor Carol Davila founded The National School of Medicine and Pharmacy, the forerunner of the University of Medicine and Pharmacy ‘Carol Davila’ in Bucharest. Along time, many famous people in medicine have started their careers from here and they have left an everlasting impression in the history of Romanian education and even universal science.

Between 1–7 October, the opening of the 2009–2010 academic year concurred with the celebration of 152 years since the beginning of the University of Medicine and Pharmacy. This event brought to our attention a series of values that were included in a few special manifestations.

One of the most important moments was the launch of academic books, amongst which ‘Surgery Manual for Students’, ‘Radiology and Medical Imaging’ and ‘Rehabilitation Compendium for Adults, Children and Elders’. The opened stands also offered a vast number and great diversity of other books in medicine and surgery that could be consulted and bought.

Because the university treasures erudition, worthy doctors have been awarded the title of ‘Doctor Honoris Causa’ of the University of Medicine and Pharmacy ‘Carol Davila’. They are Prof. Dr. Kypros Nicolaides from  King's College Hospital, London and Prof. Dr. Eliot Sorel, Member Woodrow Wilson Council at WWICS, Senior Advisor IRDL of WWICS, GWU School of Medicine and School of Public Health Founder Conflict Management Section WPA, Washington, D.C

Students also brought their fair share of contribution to this event by organizing many activities such as the Student Project Fair, the photography exhibition of ‘Photomed’, the Open Doors Days of the Society of Students in Medicine Bucharest (SSMB). ‘The Student Project Fair achieved the greatest popularity of all’, according to the SSMB spokesperson, ‘and has become the most successful manifestation of this type in the history of SSMB. The main hall of the faculty was crowded with big boards filled with posters, flyers, photographs and other materials used in the projects, each accompanied by a representative ready to explain how things worked. There was also a registration stand for new volunteers, for those who were convinced to join by resonating with the goals of the projects presented. And they were quite a few, to our joy.’

Through this contribution, the medical student proves that his or her ability to efficiently target intimate energies to study hard, have fun and involve in a lot of extracurricular activities without expecting to receive any award but self'content. This can only mean that the student has passion and devotion to what he does.

And if we agree that work makes a man noble and passion gives that man a special aura, then one can say, without any hesitation, that the doctors and medical students who work side by side are nobles invested with this title by only their hard work, day after day.

Provided with this opportunity, we would like to thank all the people who made this event possible and successful, hoping to have another great collaboration between doctors, faculty and medical students in next year's celebration of the foundation of the University of Medicine and Pharmacy ‘Carol Davila’ in Bucharest.

